# Effects of pacing strategy on metabolic responses to 2-min intense exercise in Thoroughbred horses

**DOI:** 10.1038/s41598-024-69339-x

**Published:** 2024-08-07

**Authors:** Kenya Takahashi, Kazutaka Mukai, Yusaku Ebisuda, Fumi Sugiyama, Toshinobu Yoshida, Hideo Hatta, Yu Kitaoka

**Affiliations:** 1 Department of Sports Sciences, Graduate School of Arts and Sciences, The University of Tokyo, 3-8-1, Komaba, Meguro-Ku, Tokyo, 153-8902 Japan; 2 Sports Science Division, Equine Research Institute, Japan Racing Association, Tochigi, Japan; 3 Department of Human Sciences, Kanagawa University, 3-27-1 Rokkakubashi, Kanagawa-Ku, Yokohama, Kanagawa 221-8686 Japan

**Keywords:** High-intensity exercise, Metabolomics, Pacing strategy, Skeletal muscle, Thoroughbred, Biochemistry, Biomarkers, Endocrinology

## Abstract

Evidence suggests that positive pacing strategy improves exercise performance and fatigue tolerance in athletic events lasting 1–5 min. This study investigated muscle metabolic responses to positive and negative pacing strategies in Thoroughbred horses. Eight Thoroughbred horses performed 2 min treadmill running using positive (1 min at 110% maximal O_2_ uptake [V̇O_2_max], followed by 1 min at 90% V̇O_2_max) and negative (1 min at 90% V̇O_2_max, followed by 1 min at 110% V̇O_2_max) pacing strategies. The arterial-mixed venous O_2_ difference did not significantly differ between the two strategies. Plasma lactate levels increased toward 2 min, with significantly higher concentrations during positive pacing than during negative pacing. Muscle glycogen level was significantly lower at 1 and 2 min of positive pacing than those of negative pacing. Metabolomic analysis showed that the sum of glycolytic intermediates increased during the first half of positive pacing and the second half of negative pacing. Regardless of pacing strategy, the sum of tricarboxylic acid cycle metabolites increased during the first half but remained unchanged thereafter. Our data suggest that positive pacing strategy is likely to activate glycolytic metabolism to a greater extent compared to negative pacing, even though the total workload is identical.

## Introduction

Pacing strategy significantly affects competition outcomes in athletic events^1^. In competitive events lasting 1–5 min duration, positive (fast-start or all-out) pacing strategy leads to better performance outcomes and fatigue tolerance^2–6^. The explanation for this effect has been proposed that it is linked to changes in anaerobic (high-energy phosphate and glycolytic) energy supply during the events^3,4,7^. Previous studies have investigated the effects of pacing strategies on aerobic and anaerobic contributions to energy production, primarily based on indirect indexes of muscle metabolism, such as respiratory gases and specific metabolite concentrations in the blood^4,7–9^. Among the various metabolites whose concentrations are altered during exercise, blood lactate is the most widely used indicator of exercise intensity^10^. However, reportedly, the patterns of change in blood and muscle lactate concentrations did not coincide, even when running at a constant pace^11^. Thus, further insights into muscle metabolism are limited, resulting in critical knowledge gaps. The application of metabolomics techniques together with biochemical analyses may enable us to better understand energy metabolism in skeletal muscles.

Thoroughbred horses possess extraordinary physiological traits, such as high maximal O_2_ uptake (V̇O_2_max)^12^ and muscle glycogen content^13,14^, allowing them to sustain high-intensity exercise performance for 1–3 min^15^. These remarkable athletic characteristics make Thoroughbreds suitable for elucidating metabolic responses to high-intensity exercise with different pacing strategies. In this study, we investigated metabolic responses to positive and negative pacing strategies during 2 min of intense exercise in Thoroughbred horses. To test the hypothesis that glycolytic energy production changes depending on pacing strategy, the respiratory gas, blood, and muscle samples were analyzed through metabolomics and biochemical analyses.

## Methods

### Animals and ethical approval

Eight Thoroughbred horses (three geldings and five females; age: 4–8 years old; bodyweight: 515 ± 60 kg; V̇O_2_max: 163 ± 10 mL/kg/min) were used in this study. The experimental protocols were reviewed and approved by the Animal Welfare and Ethics Committee of the Japan Racing Association Equine Research Institute (approval number: 23-27). All experiments were performed and reported in accordance with our institutional guidelines and the ARRIVE guidelines. All incisions for catheter placement and muscle biopsies were performed under local anesthesia using lidocaine. All efforts were made to minimize animal suffering.

### Preliminary incremental exercise test

Incremental exercise tests were conducted 1 week before high-intensity interval exercise. The procedure for the incremental exercise test, including O_2_ consumption measurements, has been previously described^16,17^. Briefly, the horse was warmed up by trotting at 4 m/s for 3 min, after which, an open-flow mask was fitted, and the horse began exercising at a 6% incline for 2 min each at 1.7, 4, 6, 8, 10, 12, and 13 m/s until it could not maintain its position at the front of the treadmill with verbal encouragement.

The horses wore an open-flow mask on the treadmill, through which, a rheostat-controlled blower drew air. Air flowed through a 25 cm diameter tubing and across a pneumotachograph (LF-150B, Vise Medical, Chiba, Japan) connected to a differential pressure transducer (TF-5, Vise Medical, Chiba, Japan). O_2_ and CO_2_ concentrations were measured using O_2_ and CO_2_ analyzers (FC-10 and CA-10, Sable Systems International, North Las Vegas, NV, USA), and the gas analyzer outputs for the final 30 s of each step were recorded on personal computers using commercial hardware and software (DI-720 and Windaq Pro + , DATAQ, Akron, OH, USA), with sampling at 200 Hz.

### High-intensity exercise

**Figure 1:**
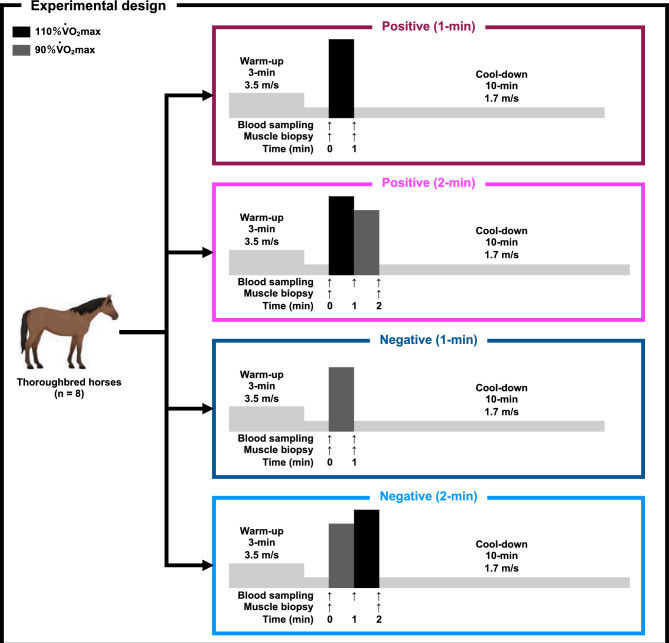
Schematic overview of the experiment.

A schematic of the experimental protocol is shown in Fig. 1. Using a randomized crossover design, eight Thoroughbred horses were subjected to four high-intensity exercise sessions on a treadmill with a washout period of at least 6 days between each trial. As in our previous study^18^, the washout period was set to allow sufficient time for glycogen replenishment in equine skeletal muscle^19^. During wash-out period, the horses were pastured in a yard and walked for 1  h/day in a walker without treadmill exercise. In the positive pacing strategy, horses exercised at 110% V̇O_2_max for 1 min, followed by 90% V̇O_2_max for 1 min (Positive [2 min]). In the negative pacing strategy, horses exercised at 90% V̇O_2_max for 1 min, followed by 110% V̇O_2_max for 1 min (Negative [2 min]). To collect the samples at the midpoint, horses exercised at 110 and 90% V̇O_2_max for 1 min each (Positive [1 min] and Negative [1 min], respectively). These variations in exercise intensity were selected based on a previous human study^3^. On each exercise session, the horses performed a warm-up exercise at 3.5 m/s for 3 min, followed by 1.7 m/s for 1 min, and a cool-down exercise at 1.7 m/s for 10 min. The heart rate was recorded using a commercial heart rate monitor (RC3 GPS, Polar, Kempele, Finland), and the mean heart rate was calculated for the final 30 s of each session.

### Blood and muscle sampling

Before leading a horse onto the treadmill, an 18-gauge catheter (Surflow, Terumo, Tokyo, Japan) was placed in the horse’s carotid artery, and an 8-F introducer (MO95H-8, Baxter International, Deerfield, IL, USA) was placed in the jugular vein. A Swan-Ganz catheter (SP5107U, Becton, Dickinson and Company, Franklin Lakes, NJ, USA) was passed via the jugular vein so that its tip was positioned in the pulmonary artery. Arterial blood samples were drawn from the 18-gauge carotid catheter and mixed-venous blood samples from the tip of the Swan-Ganz catheter at timed intervals into heparinized syringes for the final 30 s of each exercise, and were stored on ice until measured immediately following the experiment. Blood samples were analyzed with a blood gas analyzer (ABL800 FLEX, Radiometer, Copenhagen, Denmark) and, for O_2_ saturation (SO_2_) and concentration (CO_2_), with a hemoximeter (ABL80 FLEX-CO-OX, Radiometer, Copenhagen, Denmark). Following measurement of blood gases and oximetry, the blood was sampled for plasma lactate concentration with a lactate analyzer (Biosen S-Line, EKF-diagnostic GmbH, Barleben, Germany) after being centrifuged at 1740 × *g* for 10 min. The Swan-Ganz catheter in the pulmonary artery was connected to a cardiac output computer (COM-2, Baxter International, Deerfield, IL, USA) so that its thermistor registered pulmonary arterial temperature, which was recorded at each blood sampling and used to correct the blood gas measurements. Two horses were excluded due to errors in blood gas analysis.

Muscle samples were taken from each horse prior to the first of four trials and immediately after each exercise session. Muscle biopsy sampling site was set at one-third of the distance from the coxal tuber on an imaginary line drawn from the coxal tuber to the root of the tail. After shaved and aseptically prepared, the skin was locally anesthetized by subcutaneous injection of 0.5 ml of 2% lidocaine (Sandoz K.K., Tokyo, Japan). Muscle biopsy samples were obtained at 5 cm depth of the middle gluteal muscle using a 13 gauge × 3.9 cm co-axial introducer needle and a 14 gauge × 9 cm biopsy needle (SuperCore Biopsy Instrument, Argon Medical Devices, Plano, TX, USA). All muscle samples were immediately frozen in liquid nitrogen and stored at − 80 ℃ until analyzed.

### Determination of muscle glycogen concentration

The glycogen content was measured as previously described^20^. Briefly, the muscle biopsy sample (approximately 20 mg) was heated at 100 ℃ in 30% (w/v) KOH solution saturated with Na_2_SO_4_ until it was completely dissolved. Glycogen in the solution was precipitated on ice for 30 min after adding 99% (v/v) ethanol. The solution was then centrifuged at 10,000 × *g* for 10 min at 4 ℃. After the supernatant was discarded, the glycogen precipitate was dissolved in 1 N HCl and heated at 100 ℃ for 2 h to hydrolyze glycogen into glucose. After neutralization with 1 N NaOH, glucose concentration was determined using a glucose CII kit (Fujifilm Wako, Osaka, Japan).

### Metabolomics of skeletal muscle

Metabolomics analyses were performed by Human Metabolome Technologies Inc. (Tsuruoka, Japan). The frozen muscle specimen from four randomly selected horses (approximately 40 mg) was added to 750 μL of 50% acetonitrile/Milli-Q water containing internal standards (H3304-1002; Human Metabolome Technologies, Inc.) at 0 ℃ to inactivate the enzymes. The muscle was homogenized (thrice at 3500 rpm for 120 s) using a tissue homogenizer (Micro Smash MS100R; Tomy Digital Biology Co., Ltd., Tokyo, Japan), and the homogenate was centrifuged (2300 × *g* at 4 ℃ for 5 min). Subsequently, 400 μL of the upper aqueous layer was centrifugally filtered through the Millipore 5 kDa cutoff filter (9100 × *g* at 4 ℃ for 120 min) to remove the proteins. The filtrate was centrifugally concentrated and resuspended in 50 μL of Milli-Q water for capillary electrophoresis (CE)–mass spectrometry (MS) analysis. Metabolites were analyzed using CE-time-of-flight (TOF)-MS (Agilent CE-TOF-MS system) and CE-triple quadrupole (QqQ)-MS (Agilent CE and 6460 Triple Quad LC/MS systems; Agilent Technologies, Santa Clara, CA, USA). Cationic and anionic metabolites were analyzed using a fused-silica capillary (i.d. 50 μm × 80 cm) with a cationic buffer solution (p/n: H3301-1001; Human Metabolome Technologies) and an anionic buffer solution (p/n: H3302-1023; Human Metabolome Technologies), respectively, as the electrolyte. The CE-TOF-MS and CE-QqQ-MS data were analyzed using the automatic integration software MasterHand ver. 2.17.1.11 (Keio University, Japan) and MassHunter (Agilent Technologies), respectively.

### Statistical analyses

All data were analyzed using the MetaboAnalyst (Ver. 5.0, Metaboanalyst.com) and GraphPad Prism software (Ver. 10.2.0, Macintosh, GraphPad Software, La Jolla, CA, USA). Data normality and sphericity were confirmed using the Shapiro-Wilk test and Mauchly’s test, respectively (α > 0.05). A repeated measures two-way analysis of variance (ANOVA) (time × pacing) was performed to analyze the cardiorespiratory and blood parameters, muscle glycogen concentration, and the sum of metabolites (glycolytic and tricarboxylic acid intermediates [TCAIs]). When an interaction was found to be significant, Tukey’s multiple comparison test was performed to determine the differences between the pacing strategies and within the same pacing strategy. When the main effect of time, without an interaction, was detected, Tukey’s multiple comparison test was performed to determine the differences within the same pacing strategy. For individual metabolite data, one-way ANOVA was performed. Based on the calculated p-value of the one-way ANOVA, the adjusted p-value (false discovery rate [FDR]) was calculated using the Benjamini–Hochberg method. Following the FDR cutoff, Tukey’s honestly significant difference test was used to identify the differences among the groups. To identify metabolite changes in the context of metabolic pathways and networks in the metabolomic analysis, metabolite set enrichment analysis was performed using the Kyoto Encyclopedia of Genes and Genomes (KEGG) database^21^. Unless otherwise noted, all data are expressed as the mean ± standard deviation. Statistical significance was defined as p < 0.05 and FDR < 0.05 (cutoff).

### Ethics declarations

The experimental protocols were reviewed and approved by the Animal Welfare and Ethics Committee of the Japan Racing Association Equine Research Institute (approval number: 23-27). All experiments were performed and reported in accordance with our institutional guidelines and the ARRIVE guidelines.

## Results

### Cardiorespiratory and blood data

**Figure 2:**
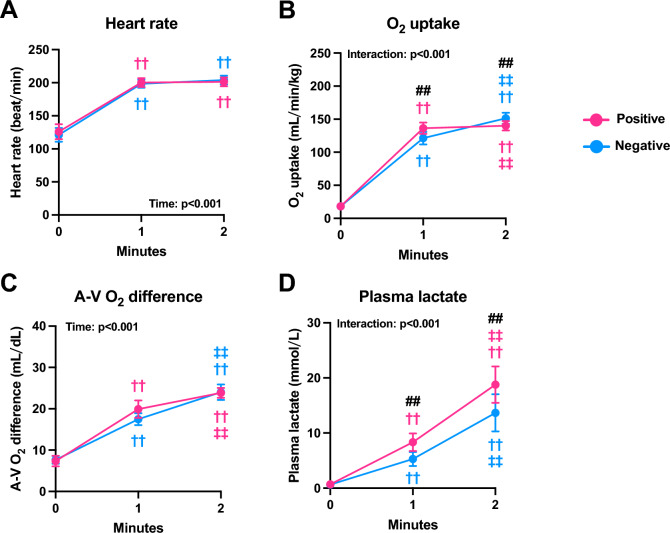
Cardiorespiratory and blood data. Heart rate (**A**), oxygen uptake/body weight (**B**), arterial-mixed venous oxygen difference (**C**), and plasma lactate concentration (**D**) during exercise employing the positive and negative pacing. Data are expressed as the mean ± standard deviation (n = 6–8). ^††^p < 0.01: vs. pre-exercise within the same pacing strategy. ^‡‡^p < 0.01: vs. 1-min within the same pacing strategy. ^##^p < 0.01: significant difference between the two pacing strategies at a given time points.

The details of the cardiorespiratory and blood data for each exercise are shown in Fig. S1. The results show the overlapping of 1 and 2 min trials of the two different pacing strategies, suggesting the successful replication of their pacing from pre-exercise to the 1 min exercise. During both 2 min trials, the heart rate increased from pre-exercise to 1 min (p < 0.001), but it did not change from 1 to 2 min (Fig. 2A). Regardless of the pacing strategy, there was a progressive increase in V̇O_2_ (p < 0.001; Fig. 2B), arterial-mixed venous O_2_ difference (p < 0.001; Fig. 2C), and plasma lactate concentration (p < 0.001; Fig. 2D) during the 2 min exercise. Compared with negative pacing, V̇O_2_ during positive pacing was higher at 1 min and lower at 2 min (p < 0.001; Fig. 2B). The plasma lactate levels at 1 and 2 min were significantly higher during positive pacing than those during negative pacing (p < 0.001; Fig. 2D).

### Muscle glycogen concentration

**Figure 3:**
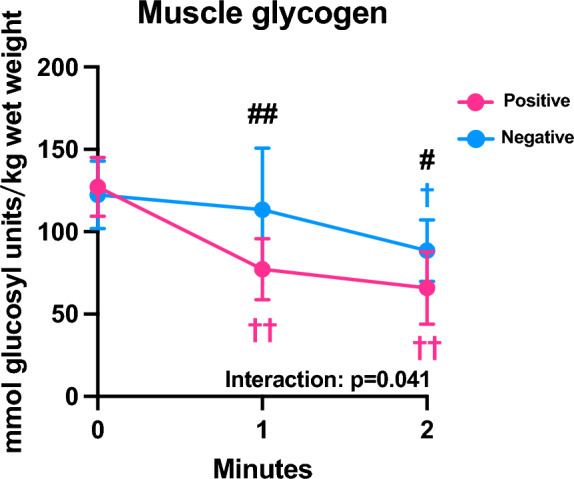
Muscle glycogen concentration. Muscle glycogen concentration during exercise employing the positive and negative pacing. Data are expressed as the mean ± standard deviation (n = 8). ^††^p < 0.01: vs. pre-exercise within the same pacing strategy. ^##^p < 0.01, ^#^p < 0.05: significant difference between the two pacing strategies at a given time points.

During positive pacing, the muscle glycogen content significantly decreased during the first half of the exercise (p < 0.001) and slightly decreased without statistical significance during the second half of exercise (p = 0.535). During negative pacing, in contrast, the muscle glycogen level was significantly lower at 2 min than at pre-exercise (p = 0.015), resulting in significantly lower glycogen concentrations at 1 min (p = 0.004) and 2-min (p = 0.046) during positive pacing than those during negative pacing (Fig. 3).

### Multivariate analysis of metabolomics

**Figure 4:**
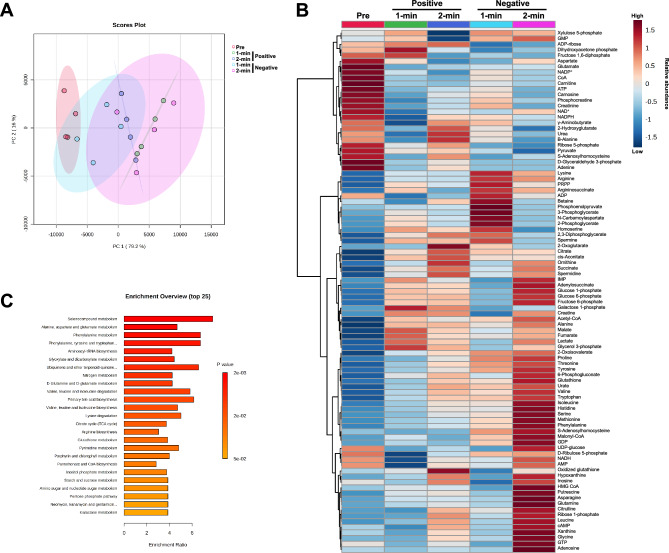
Multivariate analysis of metabolomics. Principal component analysis (PCA) (**A**), heat map of the hierarchical cluster analysis (**B**), and the top 25 enriched metabolite sets (**C**). Principal component (PC) 1 and PC2 are plotted with their 95% confidence intervals. Metabolite set enrichment analysis was performed using a database of the Kyoto Encyclopedia of Genes and Genomes (KEGG).

Of 116 targeted metabolites, 93 were detected (Table S1). The principal component (PC) analysis for the metabolomics data is shown in Fig. 4A. Although most of the 95% confidence intervals of the PC overlapped, the 95% confidence interval of pre-exercise overlapped only with 1 min of negative pacing (Fig. 4A). The heat map of the hierarchical cluster analysis of the detected metabolites is shown in Fig. 4B. The top 25 most enriched metabolite sets are shown in Fig. 4C.

### Identification of significant features in metabolomics

**Figure 5:**
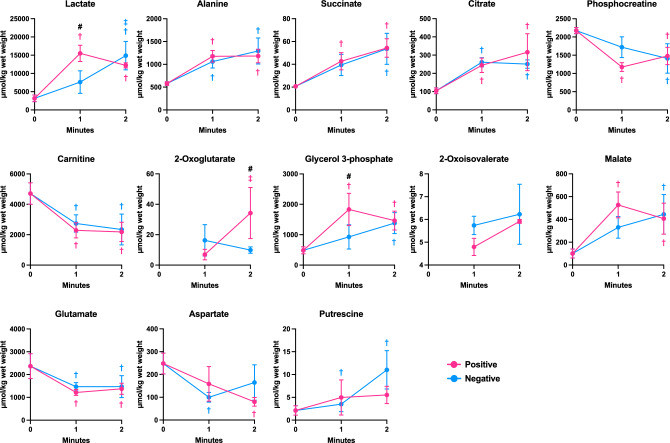
Significant features of metabolomics. Concentrations of lactate, alanine, succinate, citrate, phosphocreatine, carnitine, 2-oxoglutarate, glycerol 3-phosphate, 2-oxoisovalerate, malate, glutamate, aspartate, and putrescine during 2-min exercise using the positive and negative pacing strategies. Data are expressed as the mean ± standard deviation (n = 4). ^†^p < 0.05: vs. pre-exercise within the same pacing strategy. ^‡^p < 0.05: vs. 1-min within the same pacing strategy. ^#^p < 0.05: significant difference between the two pacing strategies at a given time points.

The results of one-way ANOVA, followed by the Benjamini–Hochberg method, showed that 13 metabolites were identified as significant features (Fig. 5; Table S2), of which 2-oxoglutarate and 2-oxoisovalerate were not detected in the samples taken before the exercise. Among the 13 significant features, lactate and glycerol 3-phosphate content at 1 min and 2-oxoglutarate content at 2 min were significantly higher during positive pacing compared with those during negative pacing (p < 0.05; Fig. 5). No further significant differences were observed between the two pacing strategies.

### Metabolite concentrations of glycolysis and mitochondria

**Figure 6:**
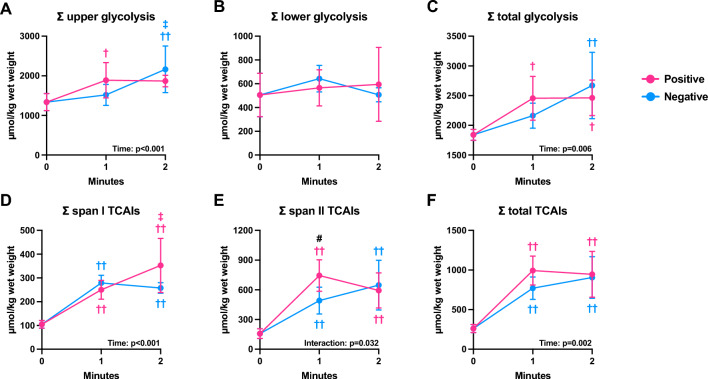
Sum of glycolytic and tricarboxylic acid cycle intermediates (TCAIs). The sum of upper glycolysis (**A**), lower glycolysis (**B**), total glycolysis (**C**), span I TCAIs (**D**), span II TCAIs (**E**), and total TCAIs (**F**) during exercise employing the positive and negative pacing. Data are expressed as the mean ± standard deviation (n = 4). ^††^p < 0.01, ^†^p < 0.05: vs. pre-exercise within the same pacing strategy. ^‡^p < 0.05: vs. 1 min within the same pacing strategy. ^#^p < 0.05: significant difference between the two pacing strategies at a given time points.

Metabolite concentrations related to high-energy phosphate, glycolysis, and mitochondria were shown in a pathway map (Fig. S2). The sum of the glycolytic and TCA intermediates, with known divisions, was calculated. During the positive pacing, Σ upper glycolysis (from glucose 6-phosphate to fructose 1,6-diphosphate) was significantly higher at 1 min than at pre-exercise (p = 0.047) but was not significantly higher at 2 min than at pre-exercise (p = 0.055) (Fig. 6A). However, no significant changes were observed between 1 and 2 min. During negative pacing, although there was no significant increase in Σ upper glycolysis from pre-exercise to 1-min, it was significantly higher at 2 min than that at pre-exercise (p = 0.008) and at 1 min (p = 0.026) (Fig. 6A). Regardless of the pacing strategy, Σ lower glycolysis (from 2,3-diphosphoglycerate to pyruvate) did not change during the 2 min exercise (Fig. 6B). During positive pacing, Σ total glycolysis (upper + lower glycolysis) was higher at 1 min (p = 0.029) and 2 min (p = 0.027) than at pre-exercise (Fig. 6C). In contrast, during negative pacing, Σ total glycolysis was higher at 2 min than at pre-exercise (p = 0.007) and tended to be higher at 2 min than at 1 min (p = 0.060) (Fig. 6C). These observations suggest that the changes in Σ total glycolysis were attributable to the changes in Σ upper glycolysis. Next, we calculated the sum of the mitochondrial TCAIs. Regardless of the pacing strategy, Σ span I TCAIs (from citrate to 2-oxoglutarate) was higher at 1 min than at pre-exercise (positive pacing: p = 0.007, negative pacing: p = 0.003), with a further increase from 1 to 2 min during only positive pacing (p = 0.037) (Fig. 6D). Although, irrespective of the pacing strategy, Σ span II TCAIs (from succinate to oxaloacetate) increased from pre-exercise to 1 min (p < 0.001); Σ span II TCAIs at 1 min was significantly higher during positive pacing than during negative pacing (p = 0.008; Fig. 6E). During both the pacing strategies, Σ total TCAIs (span I + span II TCAIs) significantly increased from pre-exercise to 1-min (p < 0.001) and remained unchanged thereafter (Fig. 6F). Collectively, despite disproportional changes in two TCAI divisions, Σ total TCAIs similarly changed in two pacing strategies.

## Discussion

### Glycolytic metabolism

Evidence has shown that positive pacing strategy improves exercise performance and fatigue tolerance in competitive events lasting 1–5 min duration in humans^2–6^. This effect has been explained by changes in anaerobic (high-energy phosphate and glycolytic) energy supply during those events^4,7^. However, there was a scarcity of direct data on skeletal muscle metabolism. To resolve this issue, we investigated muscle metabolic responses to positive and negative pacing strategies using biochemical and metabolomic techniques in Thoroughbred horses. In the present study, we found that positive pacing decreased muscle glycogen content and increased plasma lactate concentration to a greater extent compared to negative pacing. Although our data should be evaluated carefully when considering their application to humans, the current observations suggest that positive pacing strategy is likely to activate glycolytic metabolism to a greater extent compared to negative pacing, even though the total workload is identical.

### Regulation of glycolytic pathway

In addition to elucidating metabolic responses of equine skeletal muscle to different pacing strategies, this study also aimed to provide mechanistic insight, which may help understand how energy-producing pathways are regulated during exercise with different pacing strategies. In the present study, we found no significant difference in Σ total glycolysis between the two pacing strategies, despite significantly higher muscle lactate levels at 1 min during positive pacing. This may be attributable to smaller sample size used for metabolomic analysis in the present study (n = 4). Another possible explanation for this observation is the near-equilibrium reaction of glycolytic enzymes^22^, which enables lactate production with minimal accumulation of glycolytic intermediates. Specifically, the rate of overall glycolysis is determined by the flux through phosphofructokinase, which converts fructose 6-phosphate into fructose 1,6-phosphate, which is an allosteric activator that expedites another rate-limiting step in the pyruvate kinase reaction^23^, driving lactate formation without the accumulation of glycolytic products, particularly lower glycolytic intermediates.

Among the significant features, lactate and glycerol 3-phosphate were the only substrates whose levels were significantly higher at 1 min during positive pacing compared with that during negative pacing. To maintain glycolytic ATP supply, nicotinamide adenine dinucleotide (NAD^+^) regeneration is essential, as the reaction of glyceraldehyde 3-phosphate dehydrogenase requires NAD^+^ in the glycolytic pathway. Although mitochondria produce ATP and NAD^+^, their metabolism is not fully activated at the onset of exercise^24^. Hence, the working muscles increase their reliance on glycolysis for ATP production and the production of lactate and glycerol 3-phosphate for NAD^+^ regeneration. Therefore, greater increases in lactate and glycerol 3-phosphate concentrations are likely required to regenerate NAD^+^ for continuous glycolytic ATP provision.

### Oxidative metabolism

Previous studies reported faster development of V̇O_2_ during positive pacing, resulting in higher total V̇O_2_ in humans^2,3,5,25^, which suggest increased reliance on oxidative energy supply. Supporting the previous observation, we observed that V̇O_2_ at 1 min was higher during positive pacing than that during negative pacing. However, other human studies employing different pacing strategies did not observe significant differences in V̇O_2_^4,8,9^, which may be attributed to small changes in the amount of work completed during the first half of their tests. The arterial-mixed venous oxygen difference (often referred to as a-vO_2_ difference) represents the difference in oxygen content between arterial blood and mixed venous blood, indicating the oxygen extracted by tissues. Combined with cardiac output, this measurement is used to assess the amount of oxygen extracted by tissues, thereby providing an indication of oxidative metabolism. In the present study, we found that arterial-mixed venous O_2_ difference and cardiac output (Fig. S1) did not differ significantly between the two pacing strategies in horses. These observations suggest that mitochondrial oxidative ATP production occurred similarly during the exercise employing the two pacing strategies. It is, however, important to note that the measurement is limited to 1 and 2 min during exercise. Thus, we cannot rule out the possibility that dynamics of these parameters and, therefore, oxidative contribution to energy provision may differ depending on pacing strategies.

### Operation of mitochondrial TCAIs

Despite a progressive increase in arterial-mixed venous O_2_ difference toward the end of the 2 min exercise, Σ total TCAIs did not significantly change during the latter half of the two pacing strategies. Our data support the previous notion that the sum of TCAIs is not related to the mitochondrial respiration rate and that the ability to sustain flux through anaplerotic pathways is important for normal oxidative metabolism in the muscles^26^. Although various carboxylation and decarboxylation reactions regulate the carbon flux into and out of the cycle, the alanine aminotransferase (AAT) reaction, in which, pyruvate and glutamate are converted to 2-oxoglutarate and alanine, is considered the most important factor for the increase in TCAIs at the onset of exercise^27^. Supporting this notion, we observed similar changes in alanine and TCAI levels and the reciprocal changes in alanine and glutamate concentrations. The dissociation between Σ total TCAIs and arterial-mixed venous O_2_ difference is likely because flux through the AAT reaction, and thus the TCAIs pool size are determined by pre-exercise glutamate availability^28,29^.

Although TCAIs operate in two spans, this division of the cycle is only academic because the fluxes through the two pathways must be identical and regulated in a concerted manner^30^. Nonetheless, this division may explain the flux of carbon skeletons in the TCA cycle. Herein, Σ span I TCAIs progressively increased during 2 min of the positive pacing, and the negative pacing significantly increased Σ span I TCAIs only during the first half. A human study reported that citrate concentration showed a large increase and was the only TCAIs that was higher during the 2 min recovery compared with that at the end of the 5 min exercise^27^. Moreover, a different study reported an increase in muscle citrate during recovery from intense cycling exercises^31^. These observations suggest that span I substrates increase in response to a decline in energy demand. Although the precise mechanisms are not clear, the increase in span I substrates may be partly attributable to an increase in the lactate-derived carbon influx into the TCA cycle, as the muscle lactate levels of all individual values declined during the second half of positive pacing.

At 1-min of exercise, Σ span II TCAIs was significantly higher during positive pacing than during negative pacing. A previous human study reported that span II TCAIs showed a large increase compared with span I TCAIs^32^. Another human study reported that an increase in span II TCAIs is dependent on exercise intensity^33^. Altogether, it is likely that most of the anaplerotic carbon which enters the TCA cycle is directed toward increasing span II TCAIs and that their accumulation during the initial phase of exercise depended on the intensity of the exercise.

### Lactate metabolism

The lactate concentration depends primarily on the balance between its production and oxidation^34^. No significant changes were observed in the muscle lactate levels during the second half of positive pacing and the first half of negative pacing, suggesting a tighter metabolic match between lactate production through glycolysis and oxidation in mitochondria. The muscle glycogen concentration at 1 min was lower during positive pacing than during negative pacing; however, the mitochondrial TCAI content and arterial-mixed venous O_2_ differences were similar between positive and negative pacing. These findings suggest that the differences in muscle lactate concentration at 1 min between the two pacing strategies resulted primarily from differences in the rate of glycolysis.

The patterns of changes in the plasma and muscle lactate concentrations were similar during negative pacing but were different during positive pacing, particularly during the second half. Although the plasma lactate level increased during the latter half of positive pacing, the muscle lactate level did not change. This is similar to our previous observation in Thoroughbred horses that the plasma lactate levels linearly increased toward the end of the 2 min exercise at 120% V̇O_2_max, despite a smaller increase in the muscle lactate levels during the second half compared to the first half^11^. Herein, the muscle glycogen concentration did not significantly decrease during the second half of positive pacing, suggesting that the increase in the plasma lactate concentration during the latter half of the positive pacing may not result from the activation of glycogen breakdown in skeletal muscle. Given that lactate does not freely diffuse but is transported across the plasmalemma via monocarboxylate transporters^35^, an alternative explanation would be the latency of lactate to flow out from skeletal muscle and circulate in the blood.

In this study, the muscle lactate levels of all individuals declined during the second half of positive pacing and increased during the first half of negative pacing, even though the exercise intensity of each half was the same (90% V̇O_2_max). These findings may highlight the previous observation that the transition from net lactate production to consumption depends on the lactate concentration and metabolic rate in canine skeletal muscle^36^. Nevertheless, the plasma lactate level at 2 min remained higher during positive pacing. This observation corresponds to a significantly lower muscle glycogen concentration at 2 min during positive pacing, compared with that during negative pacing. Our data suggest that the muscle lactate concentration reflects a more immediate or recent glycolytic metabolism, whereas the plasma lactate levels may be more indicative of the overall glycolytic metabolism during the brief intense exercise.

### Pacing strategy in race horses

Although the optimal strategy for horses is currently poorly understood, a previous study reported a mathematical model using precise speed data from various races^37^. Their findings indicate that horses need to start strongly, reach maximum speed, and then slow down slightly throughout the race, which may be in line with our observations of changes in metabolites during different pacing strategies. However, it should be noted that our results on treadmill do not take into the consideration the effects of slopes^38^, bends^39^, and aerodynamic drafting^40^ during actual races.

### Limitations and future persperctives

Although our data provide important implications for understanding muscle metabolic responses to different pacing strategies, a limitation of the present study is that small sample size used for the analyses, especially for metabolomic analysis (n = 4). Small sample sizes can reduce statistical power, limiting the ability to detect significant differences between groups, which increases the risk of type 2 errors and necessitates cautious interpretation of our results. Another limination is that we did not evaluate exercise performance. In the present study, Thoroughbred horses performed treadmill running at a defined speed (*i.e.*, submaximal effort), which differs from practical race settings and previous studies evaluating exercise performance, where subjects exert maximal effort in the trials. Further research is necessary to investigate the metabolic responses of skeletal muscle in relation to exercise performance in competitive situations. Lastly, our data should be evaluated carefully when considering their application to humans, because the present study was carried out in Thoroughbred horses. Superior physiological characteristics in race horses, such as higher V̇O_2_max and muscle glycogen levels^12–14^, potentially create species-specific responses to exercise^41^. Whether the present findings hold true for humans should be clarified in the future studies.

## Conclusions

Our data suggest that positive pacing strategy is likely to activate glycolytic metabolism to a greater extent compared to negative pacing, even though the total workload is identical, while mitochondrial oxidative ATP production occurs similarly regardless of pacing strategy in equine skeletal muscle.

### Supplementary Information

Supplementary Figure S1.

Supplementary Figure S2.

Supplementary Tables.

## Data Availability

The data that support the findings of this study are available from the corresponding authors, upon reasonable request.
