# Supplementary figures and images for: Effects of pacing strategy on metabolic responses to 2-min intense exercise in Thoroughbred horses
Source: Sci Rep. 2024 Aug 7;14:18352. doi: 10.1038/s41598-024-69339-x (PMC11306589; doi:10.1038/s41598-024-69339-x)

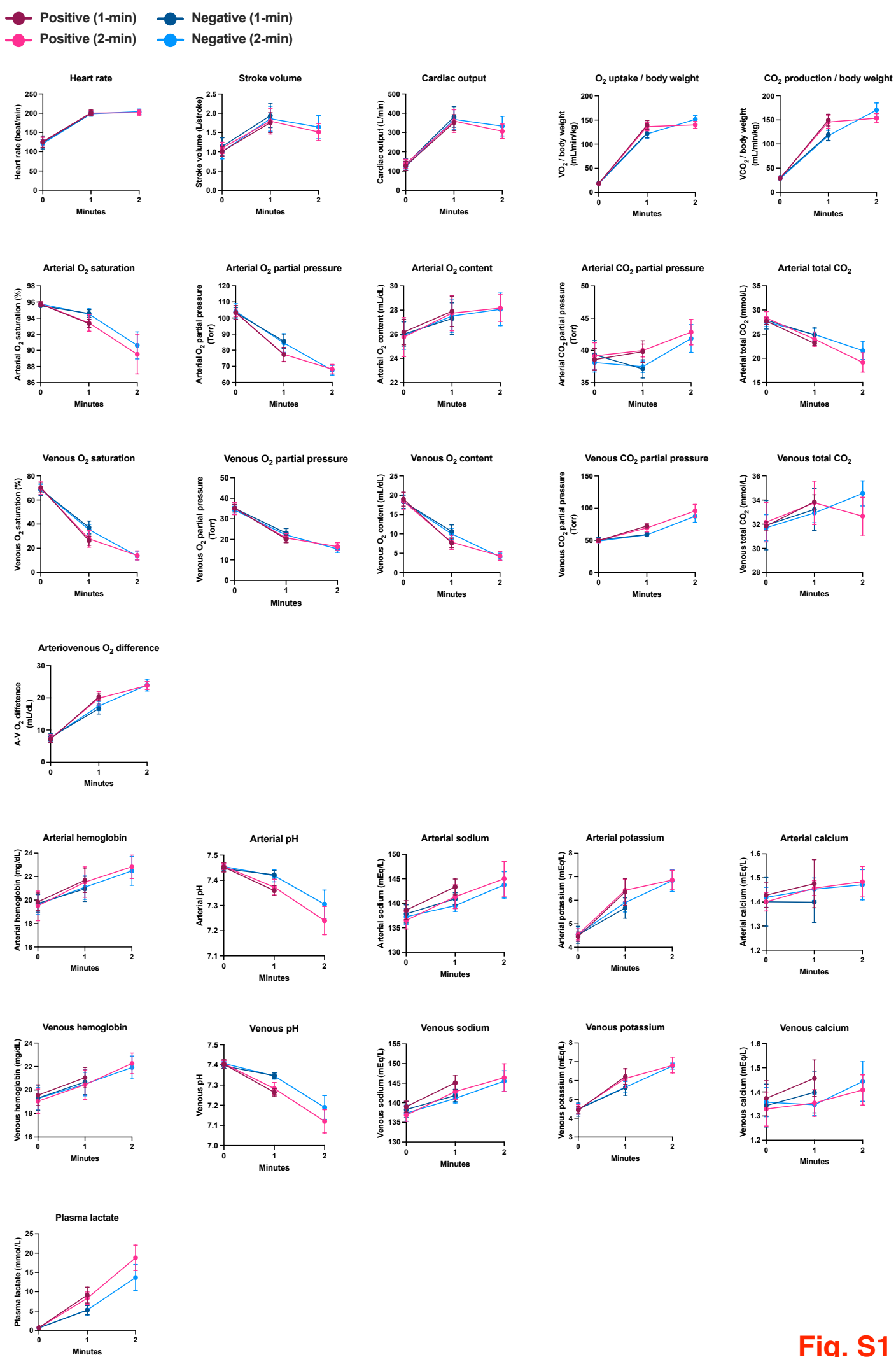

Fig. S1

Supplement: Supplementary file 1 — Supplementary Figure S1. [file 41598_2024_69339_MOESM1_ESM.pdf]

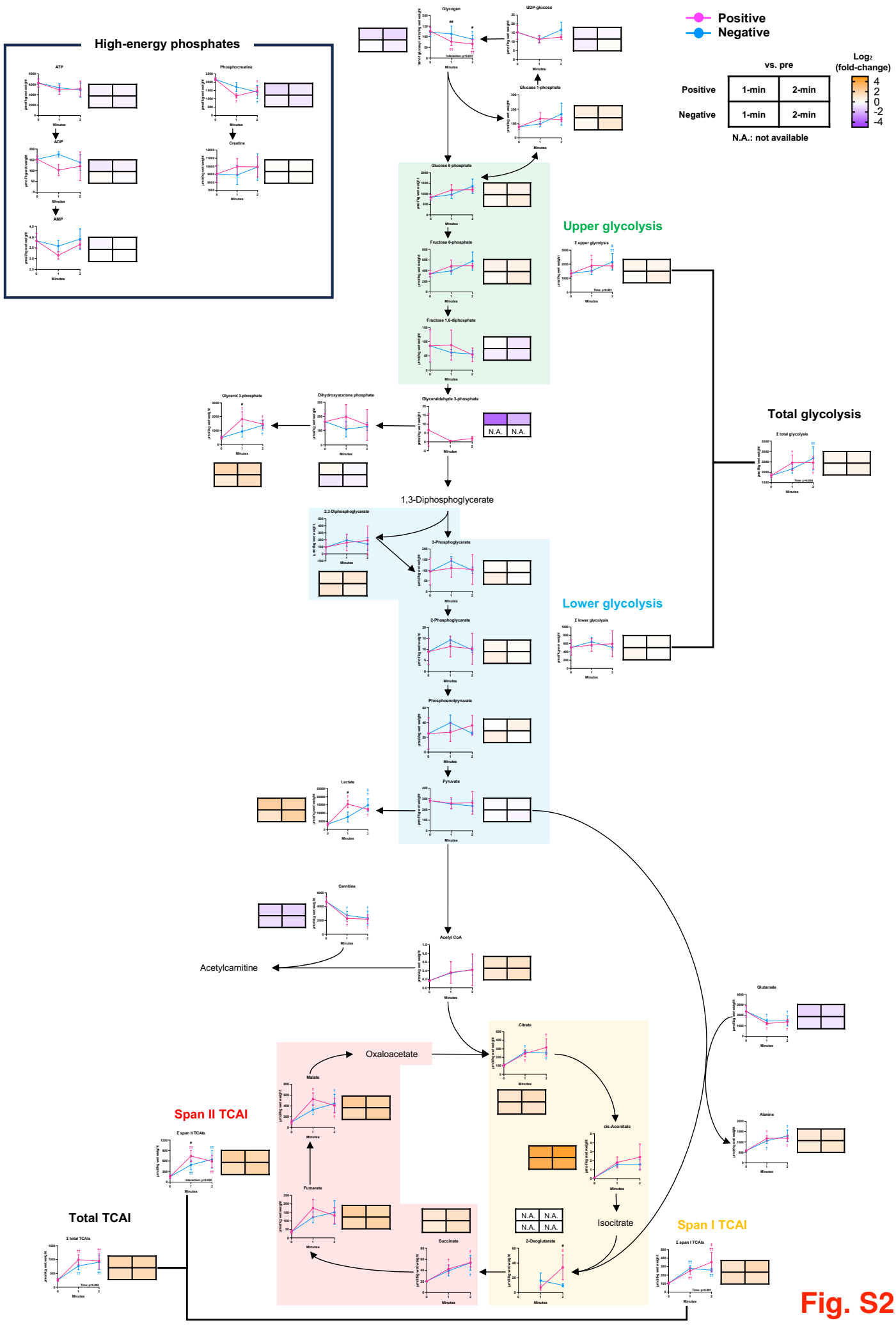

Supplement: Supplementary file 2 — Supplementary Figure S2. [file 41598_2024_69339_MOESM2_ESM.pdf]
